# Vital Life-Threatening Hematoma after Implant Insertion in the Anterior Mandible: A Case Report and Review of the Literature

**DOI:** 10.1155/2015/531865

**Published:** 2015-10-18

**Authors:** Eik Schiegnitz, Maximilian Moergel, Wilfried Wagner

**Affiliations:** Department of Oral and Maxillofacial Surgery, Johannes Gutenberg University Medical Centre, Augustusplatz 2, 55131 Mainz, Germany

## Abstract

Dental implant insertion is considered a safe and reliable surgical procedure and severe complications are seldom reported. However, we present a case of a 52-year-old patient who attended our Department of Oral and Maxillofacial Surgery, Johannes Gutenberg University Medical Center, Mainz, with spreading hematoma in the floor of the mouth and acute airway obstruction after insertion of a dental implant in the anterior mandible. The hematoma was removed and submentally drained by a silicon drainage. However, the progressive swelling of the tongue and the floor of the mouth necessitated a temporary tracheotomy for three days. The review of the literature summarizes guidelines for prevention and management of this life-threatening complication.

## 1. Case Report

**Figure 1:**
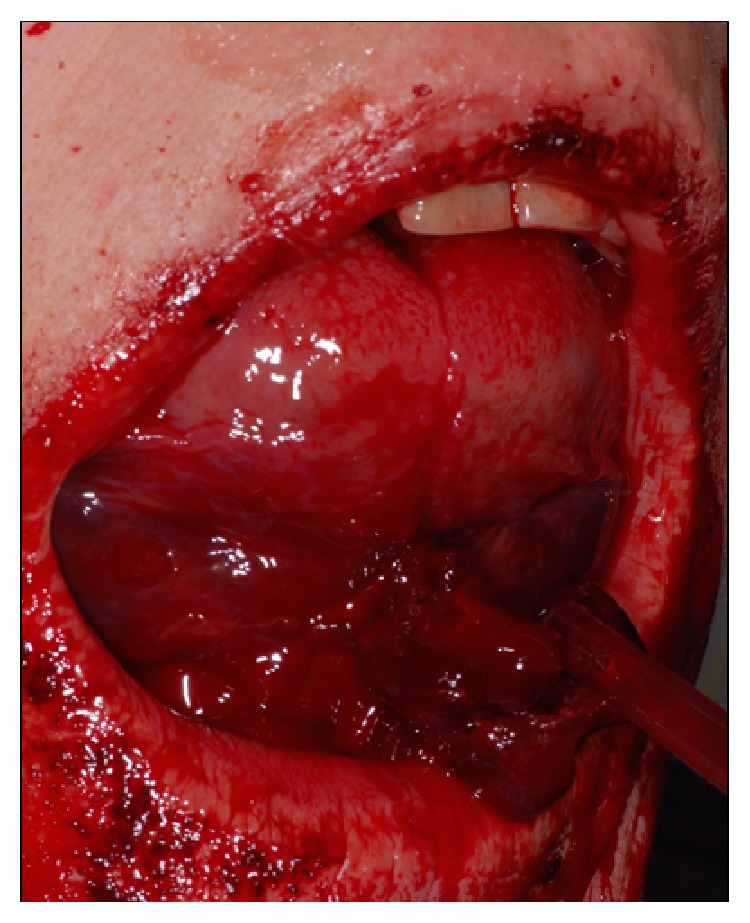
Elevated floor of the mouth with protruding tongue.

**Figure 2:**
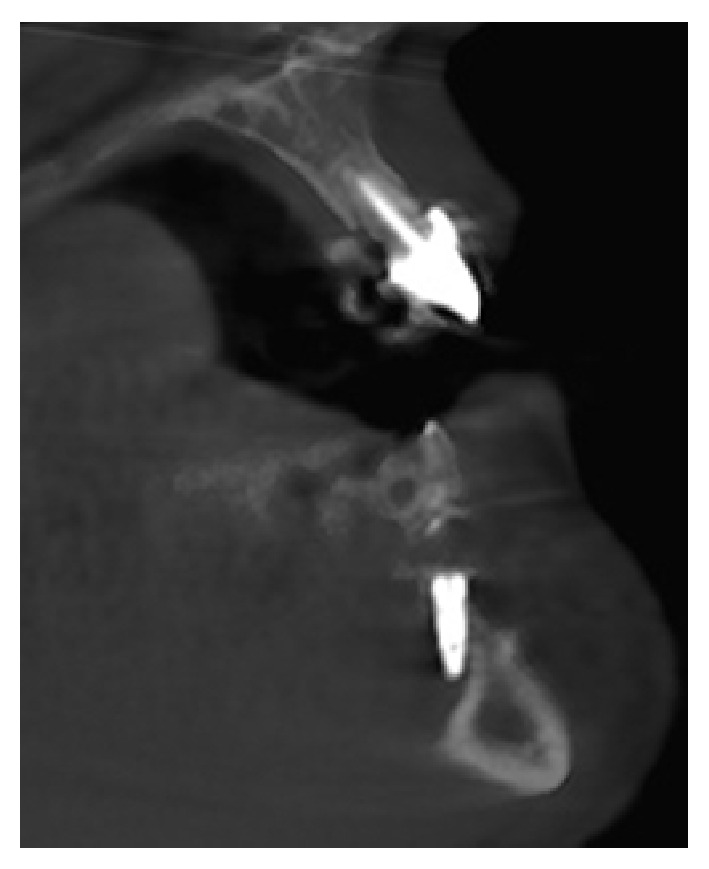
Cone beam tomography of the implant in region 32.

**Figure 3:**
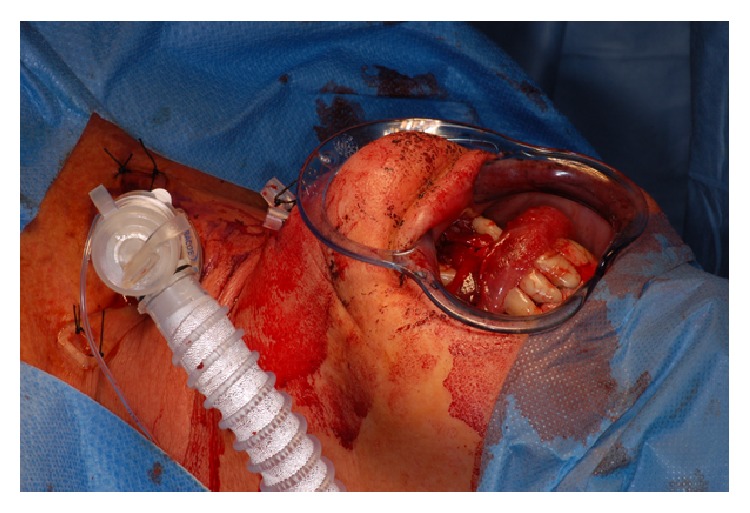
Patient postoperative with tracheotomy and extraoral drainage in the chin region.

**Figure 4:**
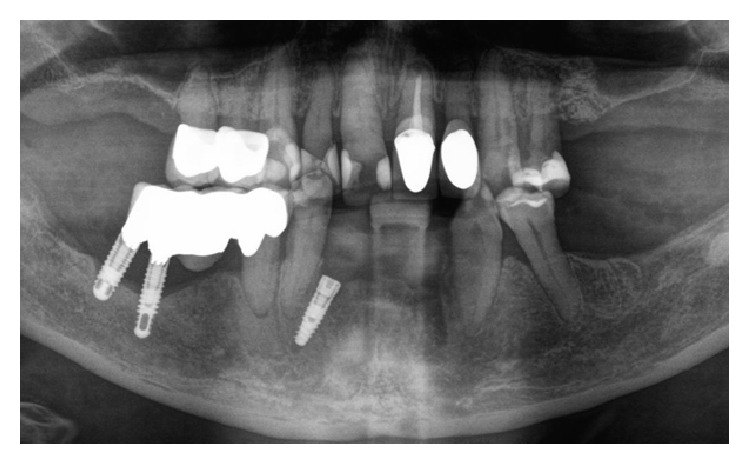
Postoperative orthopantogram.

A 52-year-old otherwise healthy woman was referred to the outpatients department of the clinic for oral and maxillofacial surgery, Johannes Gutenberg University Medical Center, Mainz, around 7.30 p.m. as emergency consultation. Two hours before the incident her dentist had performed in his private praxis immediate interforaminal implant insertion (regions 32 and 42) and guided bone regeneration after extraction of teeth 32 and 42. On clinical inspection the patient presented a dysphagia with compromised speech and swallowing. The tongue was elevated up to the hard palate by a spreading hematoma at the floor of the mouth (Figure 1). Cone beam tomography revealed an incorrectly placed implant in region 32, protruding into the deep part of the anterior floor of the mouth (Figure 2). After cone beam tomography the dysphagia progressed with fulminant respiratory distress. Therefore, the further course of this emergency case was driven by the airway management. The doctor in charge first performed a relieving incision by scalpel under local anesthesia in the anterior region. In this way, airway could be secured until the emergency team arrived at the clinic. Fiber optic intubation was carried out with difficulty but, when achieved, allowed further surgery under general anesthesia. The implant in region 32 and associated bone augmentation material was then removed from the lingual aspect of the mandible. Hemostasis was achieved using thermocoagulation. The hematoma was treated by drainage of the submental region using a silicone drain. Temporary tracheotomy for three days was indicated due to a massive swelling of the tongue (Figure 3). The postoperative course was uneventful and the swelling decreased rapidly. The postoperative orthopantogram is shown in Figure 4. Intravenous application of amoxycillin-clavulanic acid 2.2 g was performed for five days to prevent infection. Cool extra oral packs were used to reduce swelling.

## 2. Discussion

Dental implants are set worldwide with numbers in the millions, thus resembling basically a safe therapeutic option with a thorough planning and a careful operation technique as prerequisite [1–3]. However, as with any other surgical procedure, there are technical complications and biological side effects reported. In the literature, the following complications and side effects are typically described for dental implant placement: nerve damage with sensory or motor deficit, local and systemic infections, implant-related sinusitis, fractures, dislocation of the implant, implant failure, and bleeding during or after the implant placement [4, 5]. Of these, bleeding represents the complication with the highest possibility of a life-threatening consequence [6]. A literature review of the years 2000–2015 identified several case reports, which reported a severe bleeding after implantation (Table 1). Reports showed that a bleeding occurred in the vast majority after implantation in the mandible. In contrast, only one study reported an episode of bleeding after implantation in the upper jaw [7]. The main localization for life-threatening bleeding after implantation was bleeding in the area of the anterior floor of the mouth. This is attributed to an arterial trauma or injury of the periosteum or the lingual soft tissues and muscles after perforation of the lingual cortex [8]. In addition, this perforation is possible in a sloped configuration of the distal vestibular mandible. The floor of the mouth is supplied by the sublingual artery, a branch of the lingual artery, and the submental artery, as a branch of the facial artery, which both show a high degree of variability in the vascular supply and numerous anastomoses [9]. The bleeding can easily spread in the soft tissues of the floor of the mouth, including the sublingual area, resulting in an airway obstruction [9]. As it was seen in the present case, lingual perforation is an avoidable sequel of a too lingual preparation and too straight drilling sequence, if the angulated bony anatomy after resorption of the edentulous mandible is ignored by mistake. Although an interforaminal implant insertion might be a straight forward and simple procedure in the majority of cases, our case report demonstrates that it sometimes should be considered an advanced or complex action in the atrophied mandible or in cases with long-term chronic periodontitis (Figure 2) [10]. For these cases a preoperative planning with the help of a three-dimensional radiologic image (e.g., cone beam tomography) should be considered. Intraoperatively, the true width and angulation of the mandible is sometimes hard to explore by palpation; thus, partially or fully guided drilling templates which are CAD/CAM designed (computer-aided design/computer-aided manufacturing) in advance may additionally represent a helpful treatment tool [11]. Furthermore, elevating a lingual flap for better orientation and control could be in some cases helpful.

The bleeding may lead to a rapid progressive and severe swelling of the floor of the mouth with affection of the deep spaces, rapid airway obstruction, and dyspnoea as life-threatening consequence. Patients with an episode of severe bleeding may present with visible loss of blood via the oral cavity, swelling, and protrusion of the tongue and floor of the mouth. As a consequence, it comes to deficiency in swallowing, problems when speaking, and increasing dyspnoea. As first therapeutic option one may perform bidigital compression; also the insertion of tamponades or application of hemostatic agents (e.g., tranexam gel) is helpful. The patient should be calmed down to reduce hypertension in the state of anxiety. In addition, oxygen may be supplied via the nose to lessen dyspnoea stress. A rapid transport to the nearest clinic should always be made, since a suspicion of bleeding in the floor of the mouth justifies the transfer of the patient to a specialist clinic [14]. An emergency drainage of the hematoma by scalpel incision can be helpful to prevent acute airway obstruction in front of allocation to the clinic when the practitioner is trained in such a procedure. When without experience in this procedure, some authors have suggested that incision to drain the hematoma may worsen the situation [16, 17]. In the majority of cases reported in the literature a tracheotomy was temporarily necessary (Table 1). After protection of the airways a stable hemostasis may be achieved by thermocoagulation, compression, or various suture techniques and drainage of the hematoma [6]. Depending on the amount of the hematoma and the involved deep spaces, an extraoral drainage is prepared from a submental or submandibular approach as drainage backup. Dislocated and loosened implants must be removed. Postoperatively, intravenous antibiotics may be administered as prophylaxis for infection [28].

In conclusion, the risk of severe bleedings in the anterior mandible should be kept in mind. For prevention of this serious complication a detailed diagnosis and planning of surgery should be done. Risk patients (e.g., patients with anticoagulant medication or high blood pressure) should be identified in advance; the indications should be carefully reviewed and specific surgical precautions should be applied. In case of lingual perforation during implant insertion in the anterior region, the operation should be stopped or placement of a shorter implant should be considered. In unclear cases an X-ray computed tomography could be performed postoperatively. These patients should receive a prolonged follow-up and detailed information about precautions.

## Figures and Tables

**Table 1:** Case reports about vital life-threatening bleeding after implant insertion published in the years 2000–2015.

Study	Age	Region	Treatment
Givol et al., 2000 [12]	63	Anterior mandible	Surgery, tracheotomy
Niamtu, 2001 [13]	64	Anterior mandible	Tamponade, compression, and tracheotomy
Weibrich et al., 2002 [14]	60	Posterior mandible	Surgery
Boyes-Varley and Lownie, 2002 [15]	50	Anterior mandible	Surgery, tracheotomy
Isaacson, 2004 [16]	56	Anterior mandible	Surgery
Kalpidis and Konstantinidis, 2005 [17]	43	Posterior mandible	Tamponade, compression
Budihardja et al., 2006 [18]	80	Anterior mandible	Tracheotomy
Woo et al., 2006 [19]	47	Anterior mandible	Surgery, tracheotomy
de Vera et al., 2008 [20]	53	Anterior mandible	Surgery
Ferneini et al., 2009 [21]	77	Posterior mandible	Observation
Pigadas et al., 2009 [22]	71	Anterior mandible	Surgery, tracheotomy
Dubois et al., 2010 [23]	76 and 62	Anterior mandible	Case 1: surgery, tracheotomy; Case 2: surgery, tracheotomy
Hong and Mun, 2011 [7]	54	Posterior maxilla	Surgery
Felisati et al., 2012 [24]	62	Anterior mandible	Surgery, tracheotomy
Lee et al., 2012 [25]	69	Anterior mandible	Surgery
Hwang et al., 2013 [26]	53	Anterior mandible	Surgery
Sakka and Krenkel, 2013 [27]	66	Anterior mandible	Surgery
